# Response of Last Instar *Helicoverpa armígera* Larvae to Bt Toxin Ingestion: Changes in the Development and in the CYP6AE14, CYP6B2 and CYP9A12 Gene Expression

**DOI:** 10.1371/journal.pone.0099229

**Published:** 2014-06-09

**Authors:** Pilar Muñoz, Carmen López, Marian Moralejo, Meritxell Pérez-Hedo, Matilde Eizaguirre

**Affiliations:** 1 Agrotecnio Center, Universitat de Lleida, Lleida, Spain; 2 Crop Forest Sciences Department, Universitat de Lleida, Lleida, Spain; 3 Department of Chemistry, Universitat de Lleida, Lleida, Spain; Instituto de Biotecnología, Universidad Nacional Autónoma de México, Mexico

## Abstract

Bt crops are able to produce Cry proteins, which were originally present in *Bacillus thuringiensis* bacteria. Although Bt maize is very efficient against corn borers, Spanish crops are also attacked by the earworm *H. armigera*, which is less susceptible to Bt maize. Many mechanisms could be involved in this low susceptibility to the toxin, including the insect's metabolic resistance to toxins due to cytochrome P450 monooxygenases. This paper examines the response of last instar *H. armigera* larvae to feeding on a diet with Bt and non-Bt maize leaves in larval development and in the gene expression of three P450 cytochromes: CYP6AE14, CYP6B2 and CYP9A12. Larvae fed on sublethal amounts of the Bt toxin showed reduced food ingestion and reduced growth and weight, preventing most of them from achieving the critical weight and pupating; additionally, after feeding for one day on the Bt diet the larvae showed a slight increase in juvenile hormone II in the hemolymp. Larvae fed on the non-Bt diet showed the highest CYP6AE14, CYP6B2 and CYP9A12 expression one day after feeding on the non-Bt diet, and just two days later the expression decreased abruptly, a finding probably related to the developmental programme of the last instar. Moreover, although the response of P450 genes to plant allelochemicals and xenobiotics has been related in general to overexpression in the resistant insect, or induction of the genes when feeding takes place, the expression of the three genes studied was suppressed in the larvae feeding on the Bt toxin. The unexpected inhibitory effect of the Cry1Ab toxin in the P450 genes of *H. armigera* larvae should be thoroughly studied to determine whether this response is somehow related to the low susceptibility of the species to the Bt toxin.

## Introduction

Bt crops are able to produce Cry proteins, which were originally present in *Bacillus thuringiensis* bacteria. Spain is the European country with the highest number of hectares sown with Bt maize, 25.3% of the total of 97 346 hectares devoted to the maize crop, and the area has been increasing since Bt maize was introduced in 1998 [1]. The only Bt event sown is MON 810, which expresses the Cry1Ab type protein which is highly effective against the corn borers *Ostrinia nubilalis* [2] and *Sesamia nonagrioides* [3]. Maize crops in Spain are attacked by these two corn borers and by other Lepidoptera larvae that are less susceptible to Bt toxin: the leaf feeder *Mythimna unipuncta* and the maize earworm *Helicoverpa armigera* [4]. Pérez-Hedo et al [4] sugested that multiple mechanisms may be involved in the low susceptibility of these two Lepidoptera, including a high rate of toxin elimination inside the peritrophic membrane. The toxin elimination inside the peritrophic membrane could be due to rapid excretion, as occurs in *M. unipuncta* larvae [5] or to a high rate of degradation inside this space. Recently Gonzalez-Cabrera et al [6] indicated that the low levels of proteolytic enzymes involved in Cry toxin activation could be another reason for the low susceptibility of *M. unipuncta* to the Bt toxin.

Insects possess three enzyme systems providing metabolic resistance to toxins: esterases, cytochrome P450 monooxygenases and glutathione-S-transferases [7]. P450s are a multigenic superfamily of enzymes that are found in the biosynthetic pathways of ecdysteroids and juvenile hormones [8] and may be the greatest detoxification mechanism available to insects when they are exposed to a foreign agent [9]. Several authors have published excellent reviews of the roles played by these enzymes in insects [9], [10], [8], including growth, development, feeding, resistance to pesticides and tolerance to plant toxins [11]–[12]. When the resistance to insecticides is mediated by monooxygenases, it is usually due to increased detoxification or decreased activation of the xenobiotics [11] through overexpression or induction of the P450 genes [13]–[16]. The response of insects to plant allelochemicals and other xenobiotics is also mediated by monooxygenases [17]–[20].

In phytophagous caterpillars high P450 gene expression is also related to the periods of active feeding [10], [8], [21]. However, caterpillars do not feed continuously during each larval instar but show different phases that could be related to the rise and fall of the P450 expression during the instar reported by Agosin [9]. Moreover, last instar larvae undergo a phagoperiod that lasts a few days, followed by a phase of cessation of feeding and a massive purge of material from the gut [22]. The cessation of feeding due to the achievement of the critical weight [23] coincides with a change of developmental programme characterized by the turning off of the corpora allata and the secretion of juvenile hormone (JH), which leads to the release of the prothoracicotropic hormone and the activation of the prothoracic glands, followed by the pupation of the insect.

Recent studies have demonstrated that *H. armigera* has developed field resistance to the Cry1Ac toxin in China [24] and to fenvalerate in cotton in Australia [25]. Also, Zhou et al [26] reported the overexpression of several P450 genes of *H. armigera* in response to xenobiotics.

Therefore, considering the possibility of *H. armigera* developing resistance to the Cry1Ab toxin of maize in field conditions, we decided to analyse the response of larvae to the ingestion of sublethal amounts of the Bt toxin with respect to feeding behaviour, level of JH and expression of the several P450 genes identified as monooxygenases responding to xenobiotics.

## Materials and Methods

### Insects

*H. armigera* larvae were originally collected with permission of the owner (Josep Piqué) from a commercial non-Bt maize field in Lleida Spain (GPS coordinates 41°46’55.48’’N, 0°31’39.3’’E) and renewed every season. Larvae were reared on a semi-artificial diet [27]. The adults were supplied with a sugar solution (10%) and maintained at 21°C and high humidity (>60%) under a 16:8 h light: dark photoperiod.

### Effects of Bt toxin on larval development

Newly moulted caterpillars of 6th instars (L_6_0) were selected and provided with semi-artificial diets containing 9% lyophilized maize non-Bt (non-Bt diet) or Bt (Bt diet) maize leaves and 3% maize flour (all percentages refer to wet prepared diet) [28]. Every day until pupae or death, larvae (24 larvae in each type of diet) were changed to a clean box, and the larvae, ingested food and frass produced were weighed. The assimilation of ingested food (digestibility) and the ability to convert ingested and digested food into growth were evaluated by analysis of covariance [29]. The assimilation of food was examined by adjusting the amount of frass produced with food intake as covariate. The ability to convert ingested food into growth was examined by subtracting the weight of frass from the weight of ingested food and using the result as a covariate for larval weight gain [30]. Moreover, the food intake of the larvae fed on the Bt or non-Bt diet for all the instars was compared with the food intake of larva fed on a Bt diet for three days and then with the non-Bt diet for the rest of the instar.

The duration of the instars and the weight of the pupae were also recorded. A one-way ANOVA was used to analyse the effects of the Bt and non-Bt diet on pupal weight with the JMP statistical package [31]. In cases of significant differences the least significant difference Student t-test was used. Mortality and pupation were analysed using the normal approximation of the binomial test.

### Hemolymph collection

Hemolymph was extracted from eight live larvae fed on the Bt diet and eight fed on the non-Bt diet on the first and third day of the sixth instar (L_6_d1, L_6_d3) by cutting a proleg with microscissors and collecting 40 µL from each larvae in a graduated glass micropipette.

### JH II quantification

Hemolymph was collected in a vial with methanol/isooctane (1:1, vol:vol) and methoprene as an internal standard (5 ng). The hemolymph-solvent solution was vortexed for 20s and allowed to stand at room temperature for 30 min. Then, the whole sample was centrifuged at 8500 x g for 15 min, and the isooctane phase was transferred to a new glass vial. The remaining methanol phase was vortexed again, centrifuged at 10000 x g for 30 min, and combined with the isooctane phase in the same vial. The extracts were dried under nitrogen flow down and stored a −80°C. The extracts were diluted with 100 µL of methanol/water (80:20, v/v) for immediate analysis [32]. JH II, the predominant hormone in *H. armigera* [33], was measured. Five-point calibration curves, as standard, were obtained with methanol and by spiking sample blank extract free from JHII to cover a range in both cases of 1 to 100 ng/mL, with 18 ng/mL methoprene as the internal standard. To obtain blank extracts free from JHII, L6d1 larvae were decapitated and the hemolymph was extracted five days after decapitation [3]. The instrumental parameters used were an Acquity UPLC coupled to a QqQ-MS TQD (Waters, Milford, MA), that is, a triple quadrupole mass spectrometer using the ESI, APCI, and APPI interfaces, and the system was operated under Masslynx 4.1 software. The chromatographic separation was carried out at 28°C in the isocratic mode using methanol (Waters) (80+20, vol : vol) as the mobile phase. The injection volume was 15 µl in partial loop with needle overfill. The column used was a 100 mm×2.1 mm i.d., 1.7 µm, Acquity UPLC BEH C18 (Waters) at a flow rate of 400 µL/min. A total separation of 7 min was needed.

### Tissue extraction

Midguts, the tissues with the highest expression of P450 [8], were dissected from L_6_ larvae fed one (L_6_d1) and three (L_6_d3) days in Bt or non-Bt diet and from larvae fed for three days on the Bt diet and one (L_6_d4) or three (L_6_d6) days on the non-Bt diet, immediately frozen in liquid N_2_ and stored at −80°C.

### RNA isolation and cDNA synthesis

Total RNA was isolated from five midguts pooled together using TRIzol (INvitrogen, CA, USA) following the manufacturer's instructions. The RNA was quantified and its quality was assessed by agarose gel electrophoresis and absorbance measurements at λ260/λ280 nm with the Nanodrop ND-1000 spectrophotometer. Total RNA was treated with the Turbo DNA-free DNase kit (Applied Biosystems) according to the manufacturer's protocol in order to eliminate any traces of genomic DNA.

The first-strand cDNA was synthesized from 2 µg of total RNA with random hexamer primers (50 ng/µL) and dNTPs at 65°C for 5 min, then by reverse transcription in 20-µL reactions with the SuperScript III First-Strand Synthesis System kit (Invitrogen, Carlsbad, CA, USA) following the recommended protocol. Three independent RNA preparations representing three biological replicates for each treatment were used for cDNA synthesis.

The primer sets used in this study are listed in Table 1. Three primers of two different families, with different response to allelochemicals [26] were chosen. Two of the primers tested were identical to described by Zhou et al [26], the other primer was re-designed using Genomics expression software. Specificity of the polymerase chain reaction (PCR) amplification was checked by a melt curve analysis and by sequencing the PCR products.

**Table 1:** Primers used for amplification and quantification of *Helicoverpa armigera* cDNA.

Gene	Primers	Sequence (from 5′ to 3′)	Length	T°m	Amplicon size
EF-1α	EF-1α-F EF-1α-R	GACAAACGTACCATCGAGAAG GATACCAGCCTCGAACTCAC	21 20	62	279
CYP6AE14	CYP6AE14-F CYP6AE14-R	TGTGCATTTGGCGTTGAA TCCGAGATGTGGGCGTAT	18 18	54	241
CYP6B2	CYP6B2-F CYP6B2-R	CCTGAAAGATTCTTCGCGGAGGAATTGGACAGCAGCTTCGTGATGC	24 22	70	140
CYP9A12	CYP9A12-F CYP9A12-R	ATCACCTCATAGAAGATATCC CATGTCTTTCCATTCTTGACC	21 21	59	233

cDNA was used in subsequent PCR reactions carried out in the Eppendorf Mastercycler DNA Engine Thermal Cycler PCR (Eppendorf AG, Hamburg, Germany). The reaction mixtures of 25 µL contained 2.5 µL 10x buffer, 1 µL 200 µM dNTP mix, 1 µL 10 µM of each primer (Table 1), 1U of Taq polymerase (BIOTOOLS, Madrid, Spain) and 2 µL of the cDNA solution. The initial denaturing step of 1 min at 94°C was followed by 20 cycles of 20 s at 60°C with a −0.5°C change per cycle, and 1 min at 72°C; then 30 cycles of 1 min at 94°C, 1 min at 50°C and 1 min at 72°C; the reaction was concluded with 5 min at 72°C. PCR products were separated by electrophoresis in 2% agarose gel.

### Sequencing

PCR products were cleaned and extracted with the QIAquick PCR purification kit (QIAGEN, Düsseldorf, Germany) and then sequenced using the BigDye Terminator Sequencing kit v3.1 (Applied Biosystems, Foster City, CA, USA) and the ABI-3130 capillary electrophoresis system. Sequence homologies were confirmed by a nucleotide BLAST search.

### Quantitative analysis of cytochrome p450 gene expression

The expression level of P450 cytochromes in midgut tissues was analysed by quantitative real-time PCR (q-PCR), following to the MIQE guidelines [34].

q-PCR was done using a CFX 96 system and IQ SYBR Green Supermix (Bio-Rad Laboratories, 2000 Alfred Nobel Drive, Hercules, CA 94547 USA). EF-1α was used as a reference gene to normalize the target gene expression levels among samples. q-PCR of each cDNA sample and a template-free control was performed at least in triplicate. The primer sets used in this study are listed in Table 1.

Specificity of the PCR amplification was checked by a melt curve analysis (Bio-Rad CFX Manager 3.0 software) and by sequencing the PCR products. q-PCR was run in a 25 µL reaction containing 22 µL RealMasterMix/SYBR solution, 0.5 µL each of forward and reverse primer (10 µM) and a 2 µL cDNA template using the following cycling parameters: 95°C for 5 min, followed by 45 cycles of 95°C for 30 s, 55–60°C for 30 s and 68°C for 40 s. The melting curves of amplicons were measured by taking continuous fluorescence reading while increasing temperature from 58 to 95°C with 0.5°C increments for 10 s. For each gene, a serial dilution from 10- to 1000-fold of each cDNA template was performed in order to assess the efficiency of the PCR. The relative expression levels of target genes were calculated using Bio-Rad CFX Manager Software. Values represent the mean of the different replicates ± standard error. Amplification efficiencies were compared by plotting the ΔCt values of different primer combinations of serial dilutions against the log of starting template concentrations using the CFX96 software. The Ct values were adjusted to standard curves and were normalized against the levels of EF-1α.

The transformed data (sqrt (x+0.5)) were analysed by two-way ANOVA. Results were expressed as mean expression ratio (±SE) of 1-day, 3-day, 4-day and 6-day L_6_ larvae fed with the Bt and non-Bt diet.

## Results

### Effects of Bt toxin on feeding behaviour and larval development

**Figure 1:**
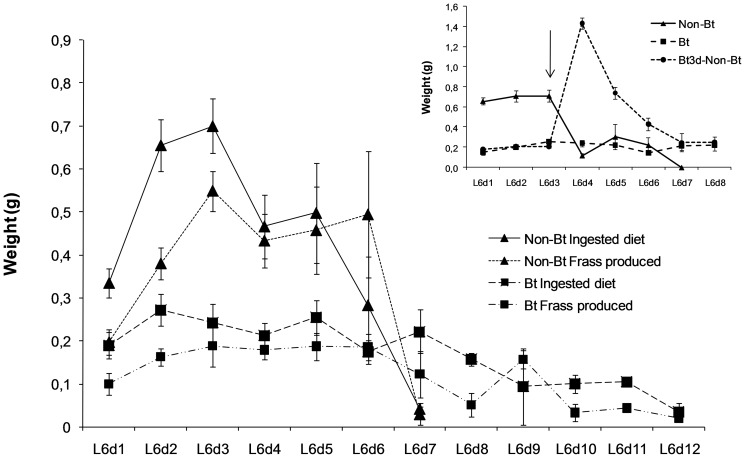
Feeding behaviour, daily mean (±SE) of ingested diet (g) and frass produced (g) of the L_6_ larvae of of *Helicoverpa armigera* fed on the Bt or non-Bt diet. Inset compares the daily ingested diet of the larvae fed for the whole last instar on the Bt or non-Bt diet with the ingested food of the larvae fed on the Bt diet for three days and then on the non-Bt diet for the rest of the instar. Arrow in the inset indicate the day the lavae were changed from the Bt to non-Bt diet.

The feeding behaviour of the larvae fed on the non-Bt diet shown in Figure 1 had three different periods. In the first period, from L_6_d1 to L_6_d3, the larvae fed actively, ingesting a greater amount of diet than the amount of frass produced. In the second period, from L_6_d4 to L_6_d5, the amount of ingested food and the frass produced were quite similar. In the third period the amount of frass produced was much greater than the amount of food ingested, signalling the purging period, which ended with the pupation of the larvae.

Feeding behaviour of the larvae fed on the Bt diet was quite different (Figure 1). The food intake was higher than the amount of frass produced until the twelfth day of the instar, except for the ninth day, and no abrupt changes in behaviour were observed. The larvae ate less than the larvae fed on the non-Bt diet but fed for a longer period. The first pupae appeared one day later than the larvae fed on the non-Bt diet (Table 2) and the majority of the larvae died without pupating. Larvae fed for three days on the Bt diet and then on the non-Bt diet for the rest of the instar displayed a sharp increase in the food intake immediately after the change of diet followed by a decrease in the feeding activity similarly to, but three days later than the larvae fed on the non-Bt diet (inset in Figure 1).

**Table 2:** Accumulated pupation (%) and mortality (%) of *Helicoverpa armigera* larvae fed on the non-Bt and Bt diet during the whole last larval instar or for three days with the Bt diet and then with the non-Bt diet during the rest of the instar (Bt3d-nonBt).

	% pupation			% mortality		
	Non-Bt	Bt	Bt3d- non-Bt	Non-Bt	Bt	Bt3d- non-Bt
L_6_d5	0.0	0.0	0	0.0	12.5	0
L_6_d6	9.1	0.0	0	4.5	16.7	0
L_6_d7	45.5	4.2	4	4.5	16.7	0
L_6_d8	72.7	8.3	16	4.5	20.8	0
L_6_d9	90.9	12.5	64	4.5	20.8	4
L_6_d10	95.5	16.7	92	4.5	37.5	4
L_6_d11		16.7	92		50.0	4
L_6_d12		16.7	92		66.7	4
L_6_d13		16.7	92		83.3	4
L_6_d14		16.7	94		83.3	4

Pupation and mortality were significantly different for the larvae fed on the Bt diet and the non-Bt (Z = −2.85, P = 0.002) and for three days on the Bt diet and then on the non-Bt diet (Z = 2.31, P = 0.001). Larvae fed on the non-Bt diet started pupation on the sixth day and finished by the tenth day of L_6_ instar (Table 2). The pupation occurred in 7.7 days on average, while in larvae fed on the Bt diet the pupation occurred in 8.8 days on average. The weight of pupae from larvae fed on the Bt diet was different (0.17 g on average), of the pupae from larvae fed on the non-Bt diet (0.32 g) and of the pupae from larvae fed 3 days on the Bt diet and then on the non-Bt diet (0.29 g) (F = 107.32; df = 2,66; P = 0.001).

**Figure 2:**
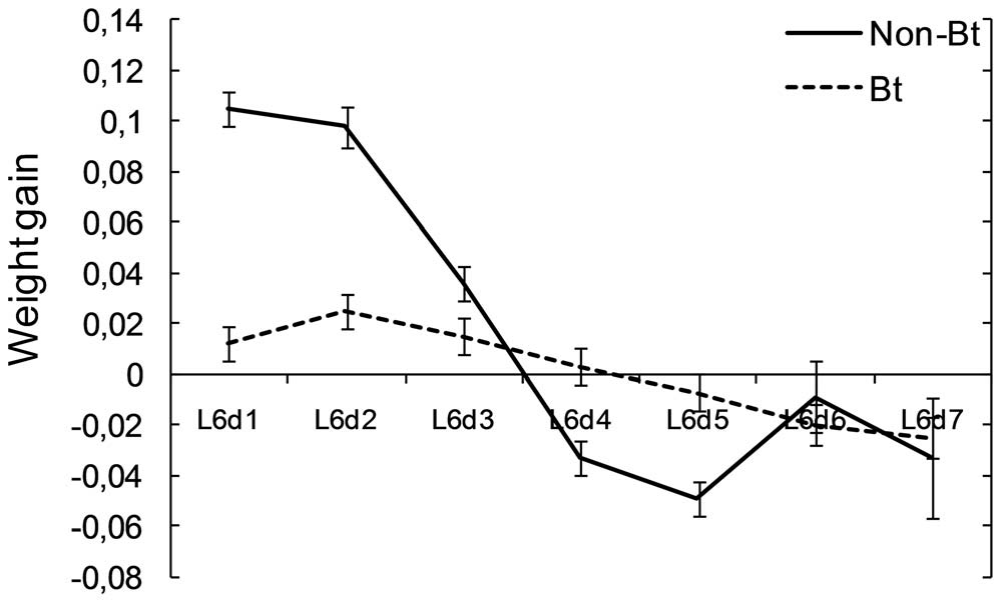
Ability to convert digested food into growth: relation between larval weight gain and the subtraction of weight of frass produced from weight of the food ingested during the last instar of *Helicoverpa armigera* larvae fed on a diet with the Bt and non-Bt leaves. Bars show least significant differences (P<0.05) for each mean in the ANCOVA of weight gain when ‘food intake-faeces’ is the covariate.

Assimilation of food examined by adjusting the amount of frass produced with food intake as covariate was influenced by the type of diet (F = 5.21; df = 1,253; P = 0.002). Two-way (diet and day) interaction was not significant (F = 0.76; df = 6, 253; P = 0.60). The influence of the diet on the weight gain significantly interacted with the day of the last instar larvae (F = 25.5; df = 6, 253; P>0.001) (Figure 2) when the subtraction food intake minus faeces was used as covariate (F = 10.07; df = 1, 253; P>0.001). The efficiency of conversion of digested food during the first two days of the last instar was significantly higher for the larvae fed on the non-Bt diet than for the larvae fed on the Bt diet. The efficiency decreased on the third day, being similar for the larvae fed on the Bt and non-Bt diet. On the fourth and fifth days of the last instar it was higher for the larvae fed on the Bt diet and on the remaining days of the instar it was similar for the larvae fed on both diets. The abrupt change in the ability of conversion of digested food of the larvae fed on the non-Bt diet on days 4 and 5 of the last instar indicated that the purge process leading to pupation had already started.

These results indicate that there was another factor that influenced the growth rate of the last instar larvae apart from the food intake or frass produced. The differences between the growth rate of the larvae fed on the Bt diet and the non-Bt diet in the first two days of the instar are probably due to the lower food intake together with the toxic effect of the Bt toxin.

### Effects of Bt toxin ingestion on the level of JH II in the hemolymph

**Figure 3:**
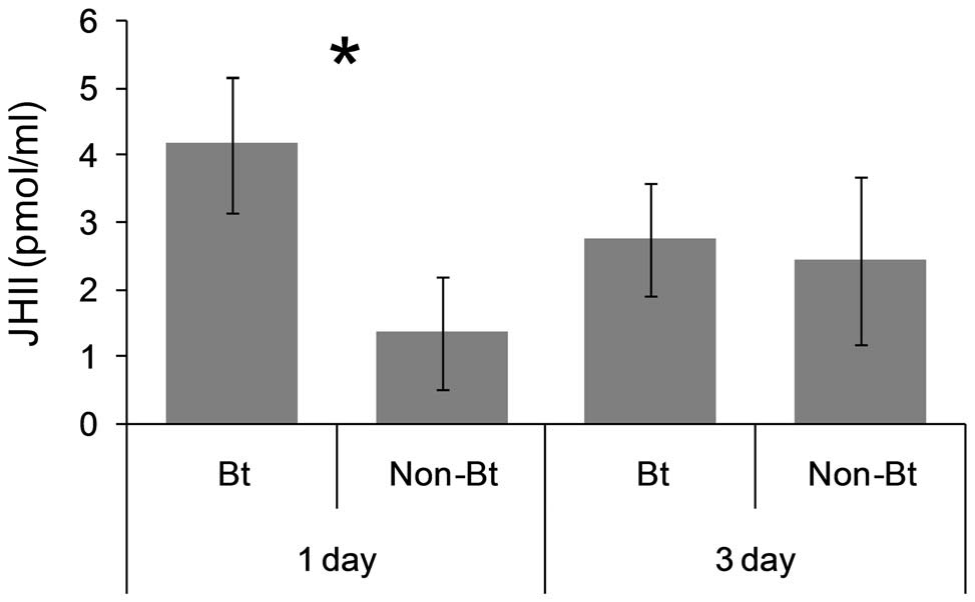
Effect of Bt toxin in the diet on the concentration of JHII in the larval hemolymph of *Helicoverpa armigera*. Larvae were fed on the non-Bt or Bt leaves for one (L_6_d1) or three (L_6_d3) days. An asterisk above the columns indicates differences between treatments, and bars indicate SE.

Figure 3 shows the concentration of JHII in the hemolymph of the first and third day of the sixth instar (L_6_d1 and L_6_d3, respectively) of larvae fed on the Bt and non-Bt diet. Concentration of JH II in the hemolymph was higher in the larvae fed on the Bt diet for one day than in the larvae fed on the non-Bt diet (F = 6.62; df = 1,14; P = 0.02) but two days later there were not differences in the JHII concentration according to the type of diet ingested (F =  0.06; df =  1,15; P = 0.81).

### Quantitative analysis of cytochrome p450 gene expression

**Figure 4:**
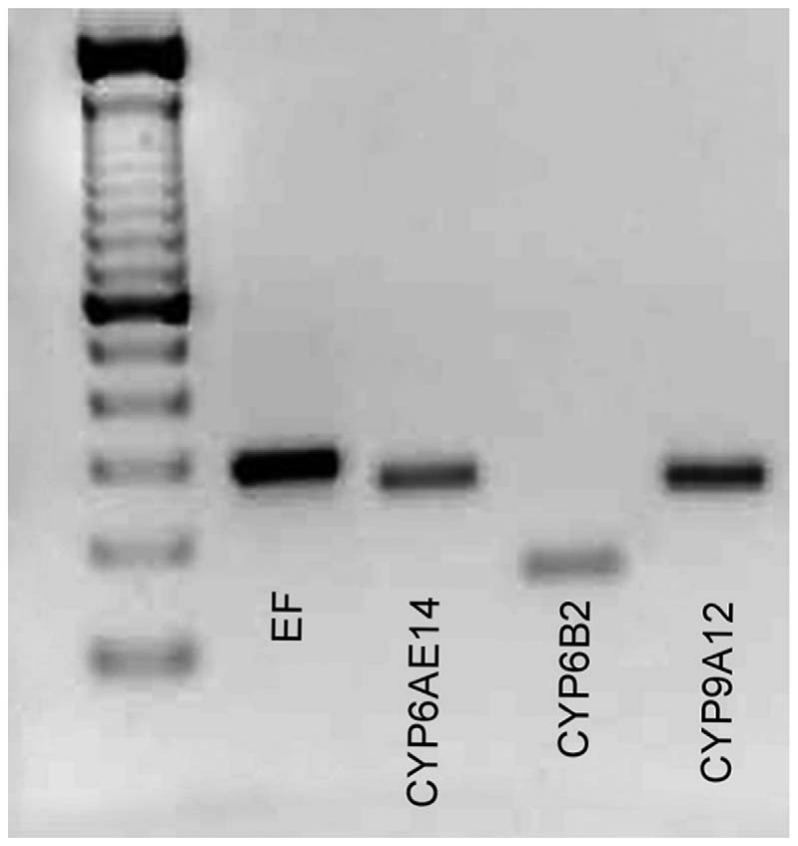
cDNA fragments produced from midgut tissues of *Helicoverpa armigera* larvae. Specific products were obtained for the elongation factor EF-1α (279bp), CYP6AE14 (241bp), CYP6B2 (140bp), and CYP9A12 (233bp).

cDNA was amplified by PCR with specific primers and separated by electrophoresis. Expected size was detected for each cytochrome (Figure 4) and confirmed by sequencing.

**Figure 5:**
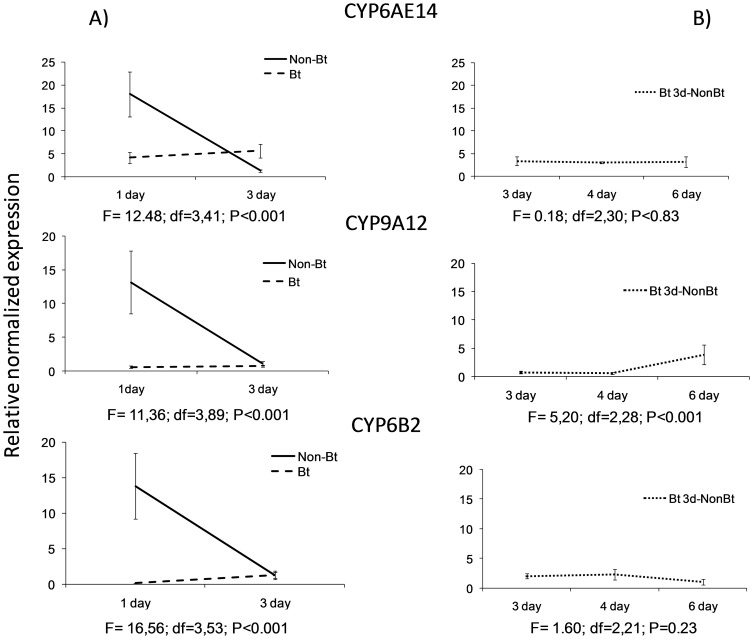
Relative normalized expression of the CYP6AE14, CYP9A12 and CYP6B2 genes in L_6_ larvae of *Helicoverpa armigera* fed A) for one (1 day) or three (3day) days on a the non-Bt or Bt diet and B) for three days on the Bt diet (day 3) and then one (4 day) or three (6 day) days on the non-Bt diet (Bt 3d-non Bt). Values represent the mean of at least three replicates ± SE.

The results of the quantitative q-PCR signalled that the expression of the cytochromes CYP6AE14, CYP6B2 and CYP9A12 was differentially affected by diet and feeding days (Figure 5).

Although the three genes compared belonged to two different families, CYP 6 and CYP 9, the response to the non-Bt diet (Figure 5A) was similar for all three. The highest expression occurred one day after feeding and the expression decreased sharply on day 3, suggesting a higher feeding activity during the first few days of the instar.

The response to the Bt toxin was quite different. Those feeding on the Bt diet almost completely suppressed the gene expression, and the expression of the CYP6AE14, CYP6B2 and CYP9A12 genes of the larvae fed on the Bt diet were 4.4, 125, and 25 times lower than those of the larvae fed for one day on the non-Bt diet. The expression of the three genes in the larvae fed on the Bt diet rose two days later but no significantly, and was higher than that of the larvae fed on the non-Bt diet only for the gene CYP6AE14 (Figure 5A). When the larvae changed the type of diet three days after feeding on the Bt diet (Figure 5B), the expressions of the genes CYP6AE14 and CYP6B2 remained low has happened in the larvae fed on the Bt diet. Only the response of the CYP9A12 gene increased after the change to the non-Bt diet in one of the repetition performed, showing a double (sample x repetition) interaction (F = 21.43; df =  2,17; P<0.001).

## Discussion

The development of the last instar of *H. armigera* followed the pattern described by Nijhout and Williams [22]. Larvae increased their weight to achieve a critical weight three days after the moult to the instar. Then, they reduced the weight drastically due to the purging, in the process leading to pupation. The achievement of the critical weight coincides with a change of developmental programme characterized by the turning off of the corpora allata and the secretion of JH, which leads to the pupation of the insect [23].

However, larvae of *H. armigera* fed on sublethal amounts of the Bt toxin showed reduced food ingestion and reduced growth and weight, which did not allow the majority of them to achieve the critical weight and pupate. This reduction of food intake, which occurs in other caterpillars in response to sublethal amounts of pesticides [35], followed by the increase of food intake that took place as a response of the change from the Bt to the non-Bt diet, coincides with the behaviour described by Slansky and Scriber [36]. These authors indicated that this behaviour may be an adaptive response to a feeding deterrent in which the reduced intake allows the larvae not to overload their detoxification and excretory system, thus avoiding death by toxicity or starvation until the change of food. This feeding behaviour may also be the consequence of the metabolic interference of the toxin with the larva's growth. Both of these phenomena may occur in the response of *H. armigera* larvae to Bt toxin ingestion.

The three P450 genes studied in the local population of *H. armigera* larvae belonged to the CYP 6 and 9 families. Some of them, such as CYP 6B2, are larval-specific [11]. The CYP 6 family is one of the most studied insect monooxygenase families in relation to insecticide resistance and plant allelochemical responses [37], [19]. CYP 9 monooxygenases have been less studied but also in relation to the response to plant allelochemicals and sometimes the response to xenobiotics [18].

Larvae fed on the non-Bt diet showed the highest CYP6AE14, CYP6B2 and CYP9A12 expression one day after feeding on the non-Bt diet, and just two days later the expression decreased abruptly. This decrease is probably related to the developmental programme of the last instar, which involves a sharp reduction in feeding after the critical weight is achieved. This change, related to JH suppression and prothoracicotropic hormone activation, may have been a consequence of P450 gene suppression or activation, because it is known that some P450 genes are involved in hormone synthesis and metabolization [10], [38]. Interestingly, the ingestion of Bt toxin led to the suppression of the three P450 genes tested, indicating that the response was not gene- or family-specific, and produced a slight increase in the JH titer in the hemolymph as was detected in the *S. nonagrioides* larvae fed on sublethal amounts of Bt toxin [3] or *Chilo suppressalis* fed on imidacloprid [39]. When larvae were changed from the Bt diet to the non-Bt diet, they recovered the feeding activity but the expression of the genes did not improve. Therefore, the suppression of the gene expression may be due not only to the reduction of the feeding activity [10], [8], [21] but also to the Bt toxin effect.

The response of P450 genes to plant allelochemicals and xenobiotics has been related in general to overexpression of the genes in the insect's resistance to insecticides, or induction of the genes when the feeding takes place [14], [17], [19], [40]. The inhibition of gene expression, although occurring in some cases [26], has been little studied. In relation to the insect's resistance to insecticides, the response of some P450 inhibitors has been studied from the point of view of the synergists of the insecticides [41], [14].

The results of the present study support the hypothesis that feeding on a Bt diet causes an suppression in the P450 expression, then reduces the feeding activity, and then the expression increases slightly and so does the feeding activity, so growth is more limited and slower. Mao et al [42] demonstrated that the larvae of *H. armigera* fed on transgenic cotton plants expressing dsCYP6AE14 showed a reduced expression level of CYP6AE14 and drastically retarded growth, so the effect achieved with the gene suppression by the dsRNA plants was somewhat similar to the effect produced by the gene suppression by the Bt toxin. It must be pointed out that the response of the P450 genes of insects to Bt ingestion has been studied very little [43].

*H. armigera* larvae have developed resistance to many insecticides [25] and to the Cry1Ac toxin in a Bt cotton in field in China [24], and have been reported to be tolerant to Bt maize in Europe [4]. The unexpected suppressive effect of the Cry1Ab toxin in the P450 genes of the CYP6 and CYP9 families of *H. armigera* larvae deserves to be further studied in order to determine whether the response to other Cry toxins is similar, whether the suppressive effect of the toxin can act as a synergist for other xenobiotics or other Cry toxins, how the strains of *H. armigera* resistant to insecticides respond to Bt toxins, and whether this response is related in some way to the low tolerance of the species to the Bt toxin.
